# Supplementary material for: DMRforPairs: identifying Differentially Methylated Regions between unique samples using array based methylation profiles
Source: BMC Bioinformatics. 2014 May 15;15:141. doi: 10.1186/1471-2105-15-141 (PMC4046028; doi:10.1186/1471-2105-15-141)
Supplement: Additional file 2 — DMRforPairs output for the comparison of A431-MCF7 and NA17018-NA17105. Please start from the HTML files in each folder. Available via the BMC Bioinformatics website. [file 1471-2105-15-141-S2.zip › 1394847754114233_MOESM2_ESM/A431_MCF7/figures/13151.pdf]

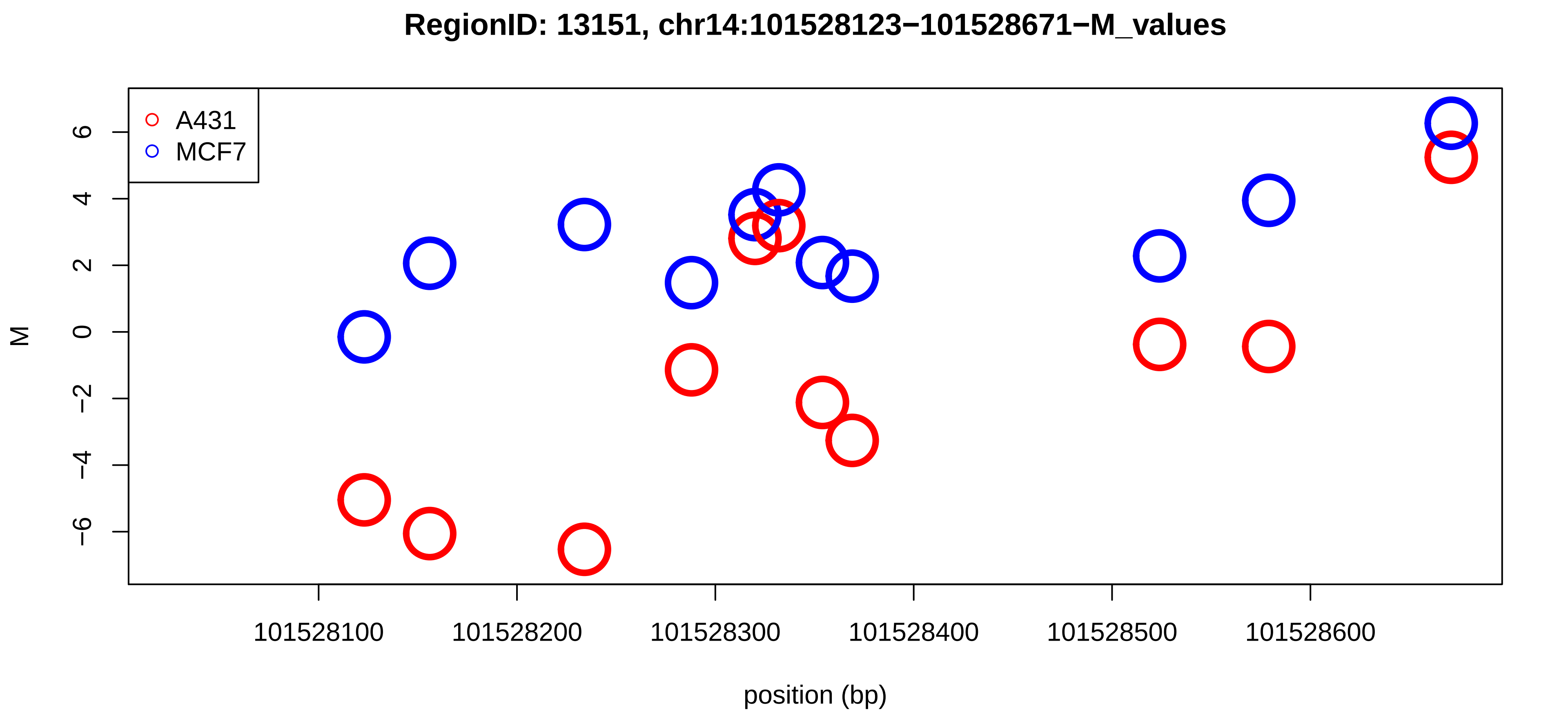
RegionID: 13151, chr14:101528123−101528671−M_values
A431
MCF7
M
6
4
2
0
−2
−4
−6
101528100
101528200
101528300
101528400
101528500
101528600
position (bp)

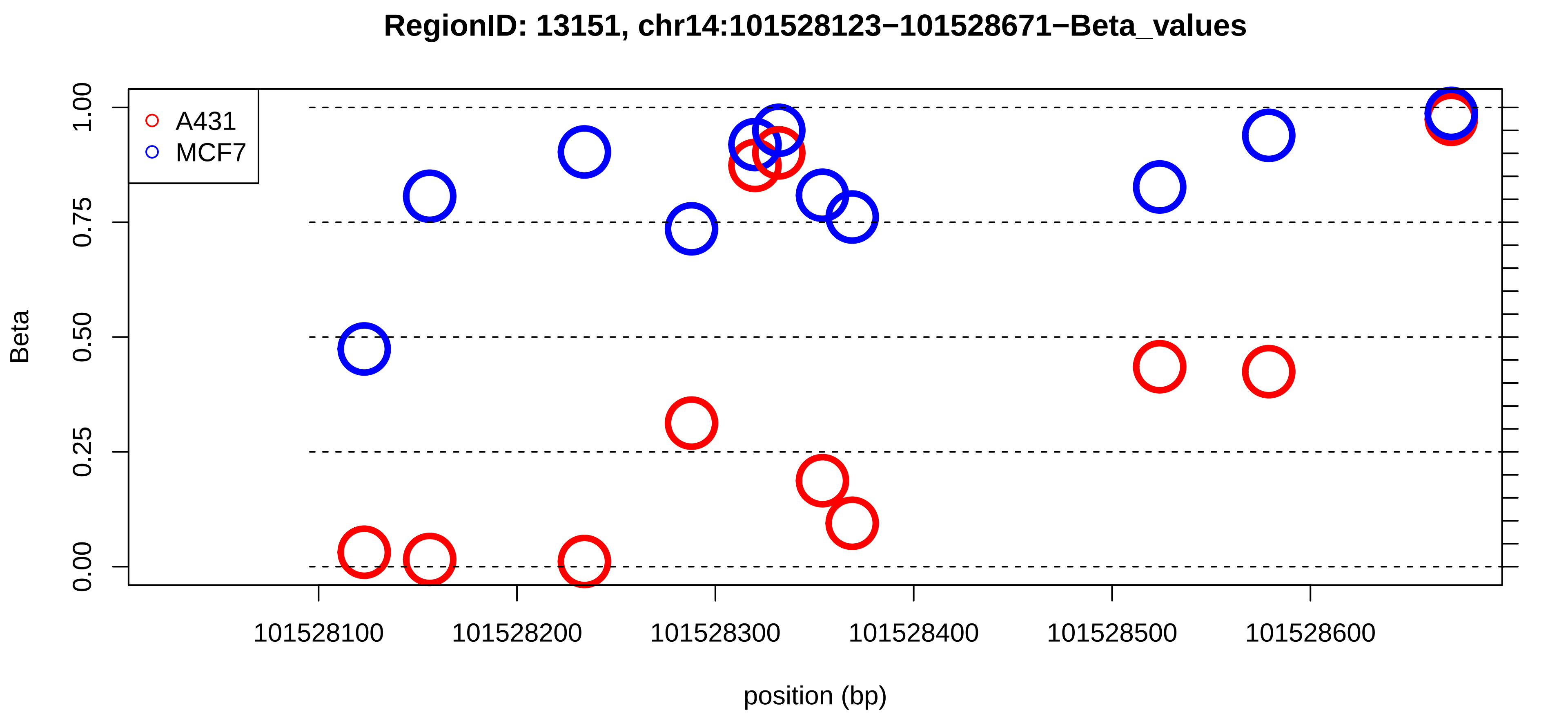
RegionID: 13151, chr14:101528123−101528671−Beta_values
A431
MCF7
Beta
1.00
0.75
0.50
0.25
0.00
101528100
101528200
101528300
101528400
101528500
101528600
position (bp)

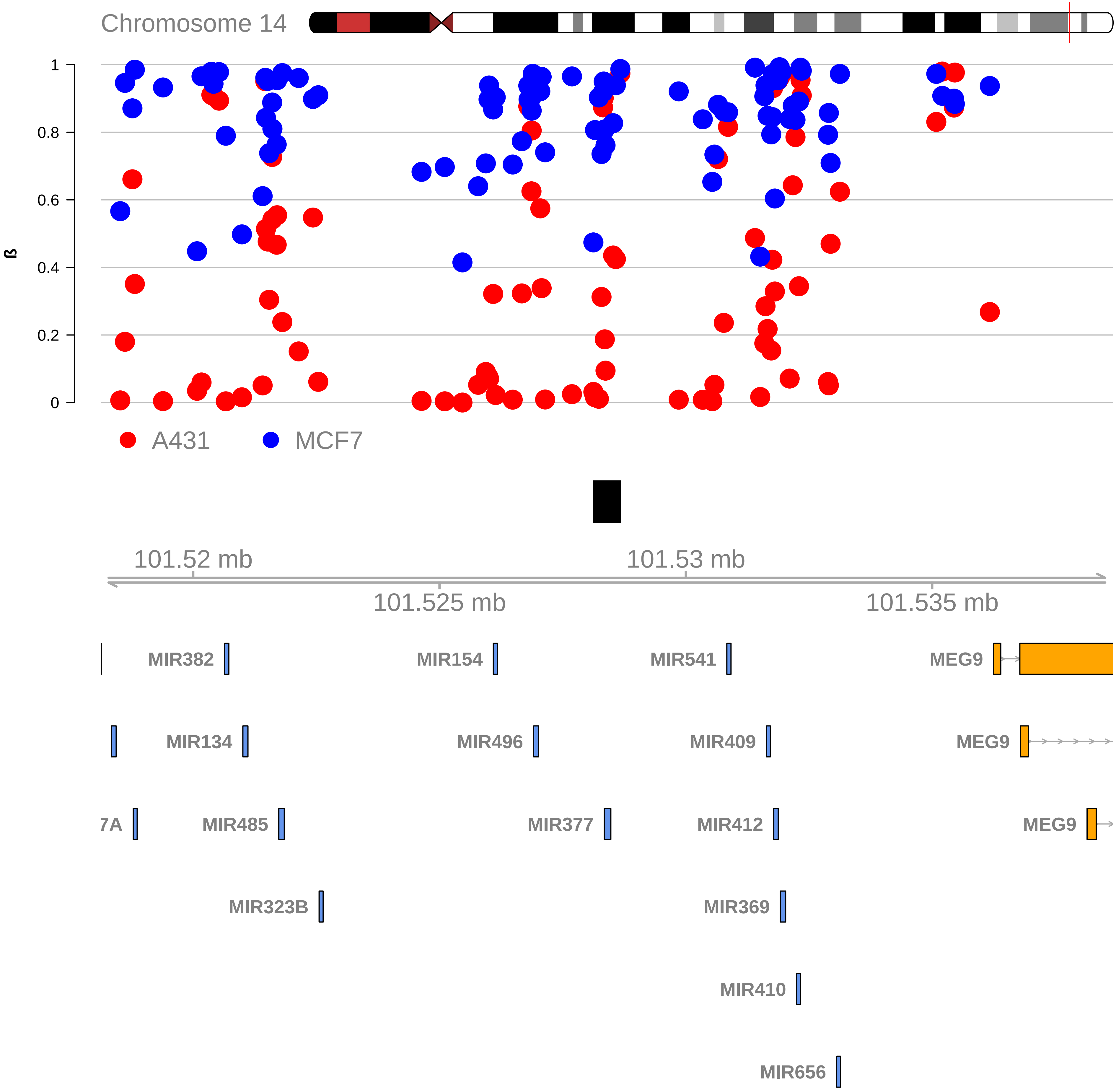
Chromosome 14
1
0.8
0.6
0.4
0.2
0
ß
A431
MCF7
101.52 mb
101.53 mb
101.525 mb
101.535 mb
MIR382
MIR154
MIR541
MEG9
MIR134
MIR496
MIR409
MEG9
7A
MIR485
MIR377
MIR412
MEG9
MIR323B
MIR369
MIR410
MIR656
